# Seroprevalence and associated risk factors of *Toxoplasma gondii* infection among slaughterhouse workers in Yangon Region, Myanmar: A cross-sectional study

**DOI:** 10.1371/journal.pone.0284352

**Published:** 2023-04-13

**Authors:** Nay Hein Sint, Ye Minn Htun, Tun Tun Win, Aye Sandar Mon, Thant Zaw Lwin, Lwin Oo Maung, Pyae Sone Win, Kaung Myat Naing, Thet Paing Zaw, Pyae Hpone Naing, Sai Nyan Lin Tun, Aung Aye Kyaw, Kyaw Wunna, Khine Khine Su, Kyaw Myo Tun

**Affiliations:** 1 Department of Preventive and Social Medicine, Defence Services Medical Academy, Yangon, Myanmar; 2 Department of Prevention and Research Development of Hepatitis, AIDS and Other Viral Diseases, Health and Disease Control Unit, Nay Pyi Taw, Myanmar; 3 Department of Biostatistics and Medical Demography, University of Public Health, Yangon, Myanmar; 4 Department of Microbiology, Defence Services Medical Academy, Yangon, Myanmar; 5 Traditional Medicine Research Division, Defence Services Medical Research Centre, Nay Pyi Taw, Myanmar; 6 Department of Anaesthesiology, Defence Services Medical Academy, Yangon, Myanmar; 7 Department of Microbiology, Military Institute of Nursing and Paramedical Sciences, Yangon, Myanmar; Kafrelsheikh University Faculty of Veterinary Medicine, EGYPT

## Abstract

**Background:**

Toxoplasmosis, having the significant consequences affecting mortality and quality of life, is still prevalent in various places throughout the world. The major gap in surveillance for *Toxoplasma gondii* infection among high-risk population, slaughterhouse workers, is an obstacle for the effective policies formulation to reduce the burden of toxoplasmosis in Myanmar. Therefore, this study aimed to assess the seroprevalence of toxoplasmosis and associated factors of seropositivity among slaughterhouse workers in Yangon Region, Myanmar.

**Methods:**

A cross-sectional study that was conducted from June to November 2020 included 139 slaughterhouse workers involving at five main slaughterhouses under Yangon City Development Committee, Myanmar. The presence of IgG and IgM anti-*T*. *gondii* antibodies in serum was detected using the *OnSite* Toxo IgG/IgM Combo Rapid Test. A face-to-face interview was also performed using pretested structured questionnaires to obtain the detail histories: sociodemographic characteristics, level of knowledge, occupational factors, and environmental factors related to *T*. *gondii* infection. Bivariate logistic regression was used to determine the factors associated with *T*. *gondii* infection.

**Results:**

Of all participants, the overall seroprevalence of anti-*T*. *gondii* was 43.9% (95% CI: 35.5–52.5%), of whom 98.4% (95% CI: 91.2–100.0%) were reactive only for IgG antibody and 1.6% (95% CI: 0.0–8.8%) were reactive for IgG and IgM antibodies. The significant factors associated with the seropositivity of *T*. *gondii* antibodies were blood transfusion history (OR: 5.74, 95% CI: 1.17–28.09), low level of knowledge (OR: 2.91, 95% CI: 1.46–5.83), contact with animal organs, muscles or blood (OR: 14.29, 95% CI: 1.83–111.51), and animals most frequently slaughtered (cattle) (OR: 3.22, 95% CI: 1.16–8.93).

**Conclusions:**

A high seroprevalence of toxoplasmosis was detected among slaughterhouse workers in Yangon Region and it raises a significant public health concern. Therefore, providing health education regarding toxoplasmosis, enforcement of personal hygiene practices in workplaces, the establishment of training for occupational hygiene, and commencement of the risk assessment and serological screening for toxoplasmosis are crucial to curtail the prevalence of *T*. *gondii* infection among slaughterhouse workers.

## Introduction

Toxoplasmosis, a cosmopolitan zoonosis, one of the neglected parasitic infections, is caused by *Toxoplasma gondii* which has been generally considered the only valid species of the *Toxoplasma* genus [1, 2]. About one-third of the worldwide population is seropositive for toxoplasmosis and it has been shown that more than 60% of some populations have been infected with *Toxoplasma* in various places throughout the world [3–5]. In the Southeast Asian population, the seroprevalence of toxoplasmosis was estimated to vary from less than 2% up to 70% [6]. Infection is often highest in areas throughout the world that are hot, humid climates and lower altitudes because the oocysts survive better in these types of environments [1, 7]. Toxoplasmosis significantly affects the health of humans, domestic animals, wildlife, and ecosystems, and is recognized as a major threat by those who rely on animal resources [8]. Currently, *T*. *gondii* infections often go underdiagnosed, underreported, and underestimated in both human and animal hosts [9].

*T*. *gondii*, is a single-celled parasite and capable of infecting most genera of warm-blooded animals including humans and livestock, as intermediate hosts [1, 2]. It is an obligate apicomplexan intracellular protozoan with a complex lifecycle consisting of a sexual cycle in its definitive hosts and an asexual cycle in its intermediate hosts [4, 10]. It can infect wild and domestic animals like cats, felids, hares, horses, rabbits, birds, and pigeons [11–17]. *T*. *gondii* infection is most common in many meat-producing animals such as cattle [16, 18–21], pigs [10–12, 14, 16–18, 20, 22], goats [2, 10, 12, 14, 18, 20, 23], sheep [2, 10, 12, 14, 20], and chicken [16]. Infection with *T*. *gondii* in sheep, pigs, and goats is higher prevalent than the infection in cattle, horses, and water buffaloes, and therefore, it is responsible for major economic losses in livestock through abortions, delivery of dead or debilitated offspring [10, 24].

*T*. *gondii* infection is also widespread in humans, an accidental host, and its prevalence varies widely depending upon the geographic location. People typically become infected through a variety of exposures: food-borne infection with ingestion of undercooked or raw meat containing the parasite in tissue cysts, consumption of food or water contaminated with oocysts, mother-to-child transmission during, or just prior to, her pregnancy, and organ transplantation or blood transfusion from an infected donor [3, 7, 24–26]. The factors such as environmental conditions, cultural behaviour, hygienic practices, cooking methods, and host immune status influenced the transmission of toxoplasmosis. The dormant form of *T*. *gondii* is found predominantly in nervous and muscle tissues in infected hosts. Acquired *Toxoplasma* infection in immunocompetent persons is generally asymptomatic and subclinical. The clinical course is usually self-limited or often seen as a mild illness characterized by fever, malaise, and lymphadenopathy [4, 25]. Ocular infection with visual loss can be occurred in rare cases. Immunodeficient patients with toxoplasmosis often present neurological signs (including headache, disorientation, drowsiness, hemiparesis, reflex changes, and convulsions) and may also have pneumonia, retinochoroiditis, and other disseminated systemic diseases [27–29].

Occupational exposure to raw meat when slaughtering animals for food could be a risk for *T*. *gondii* infection. Many seroprevalence studies of *T*. *gondii* infection among butchers, abattoir and slaughterhouses workers occupationally exposed to raw meat in some countries have been reported [19, 30–43]. In Myanmar, one of the developing countries, there were some studies on the seroprevalence of toxoplasmosis in domestic animals: goats 11.4% [44], household cats 41.30% [45], and backyard pigs 18.4% [46]. The epidemiological studies done on humans have reported varying in the prevalence of toxoplasmosis: school children 3.8–43.8% [26, 47, 48], reproductive-aged women 11.5% [49], pregnant women 30.70% [50], and refugee and migrant pregnant women 31.7% [51]. However, there is no adequate information regarding the epidemiological status of toxoplasmosis among slaughterhouse workers in Myanmar. Therefore, this study was conducted to assess the prevalence of toxoplasmosis and its associated factors among slaughterhouse workers in Yangon Region, Myanmar.

## Materials and methods

### Study design and setting

**Fig 1:**
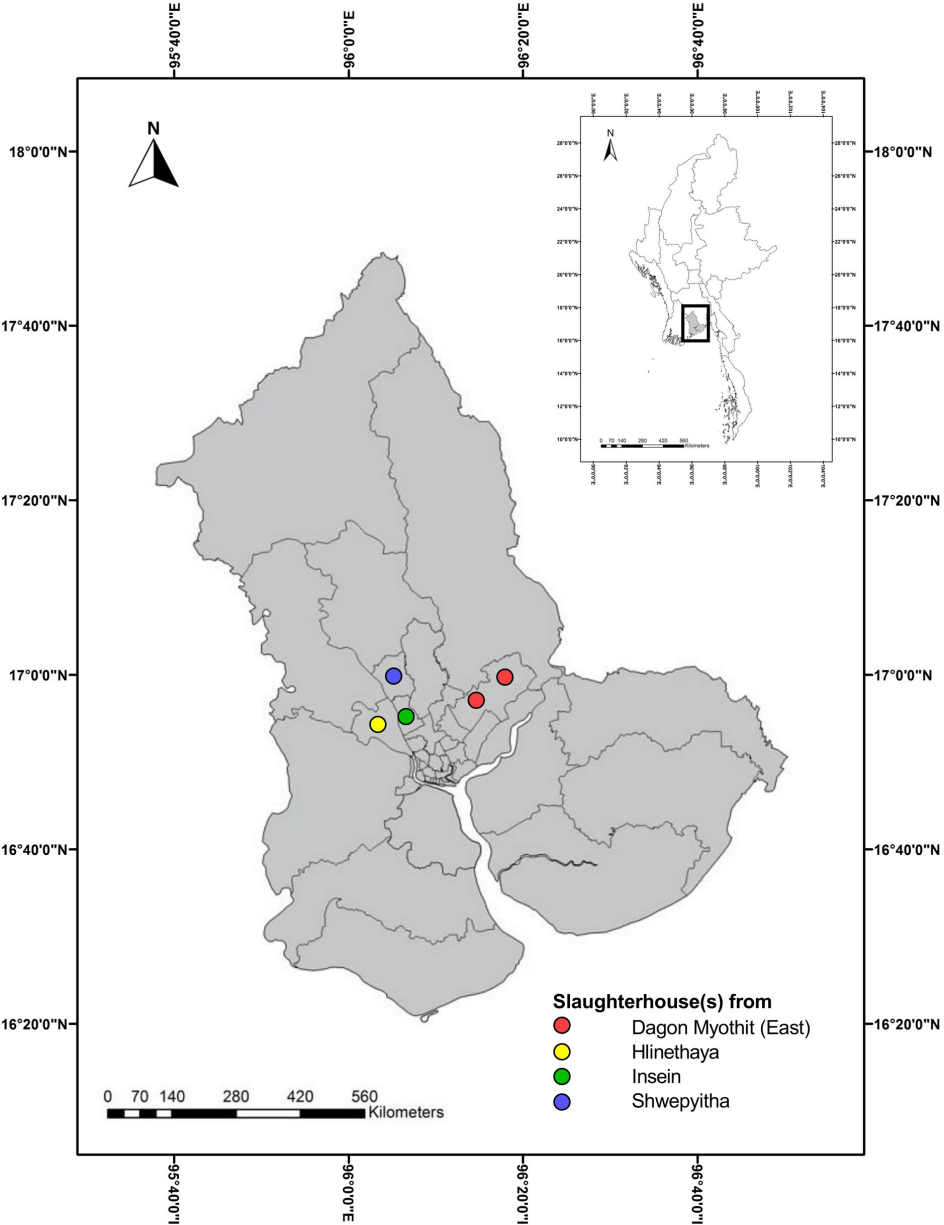
Map of the location of main slaughterhouses, Yangon Region, Myanmar (study area). This is an original figure created using ArcGIS and the link to map base layer used in creating this figure: http://geonode.themimu.info/layers/?limit=100&offset=0”.

A cross-sectional study was conducted in Yangon Region, the industrial and commercial center of Myanmar, from June to December 2020. Yangon Region sits within the wider Delta Region of the south, sharing borders with Ayeyarwady to the west, and Bago to the north and east, and resting on the Andaman Sea to the south. The total population of Yangon Region represents 14.3 percent of the total population of Myanmar, the highest in size compared with other States and Regions in the country [52]. There were five main slaughterhouses [two from Dagon Myothit (East), one from Hlinethaya, one from Insein, and one from Shwepyitha Townships] under the control of Yangon City Development Committee (YCDC) (Fig 1).

### Study population and sampling procedure

The workers involved in the slaughterhouses in Yangon Region, who were elder than 18 years and those who were with more than one year of employment duration, were eligible for the study. The sample size was estimated using the formula for single population proportion for quantitative studies [53], with the assumptions of 70% *T*. *gondii* seroprevalence in slaughterhouse workers [36], at a 95% confidence level, 5% margin of error. After adding the 10% non-response rate, a total of 139 slaughterhouse workers, were enrolled in this study. Lists of the slaughterhouse workers obtained from respective authorized persons of five slaughterhouses were used as the sampling frame. By using simple random sampling, every 28 participants were selected from four slaughterhouses and 27 participants were from one slaughterhouse.

### Data collection tools and procedures

Data were collected by face-to-face interviews using a pretested structured questionnaire. It was prepared based on the previous literature [36, 54] and modified into the context related to that study area. The questionnaire was constructed into four parts: 1) sociodemographic with 10 items, 2) knowledge about toxoplasmosis with 17 items, 3) occupational factors with 9 items, and 4) environmental factors with 8 items. There were single-response and multiple-response items in knowledge about toxoplasmosis, occupational factors, and environmental factors. The responses of knowledge about toxoplasmosis were assigned as “yes”, “no”, and “don’t know”. For the scoring system, the correct responses were scored as one point, and incorrect responses or “don’t know” were scored as zero point. The English version questionnaire was translated into the Burmese language, the local language of the study area, and then translated back to English by language and public health experts.

The *OnSite* Toxo IgG/IgM Combo Rapid Test (CTK Biotech, Inc., USA), lateral flow chromatographic immunoassay, can detect and differentiate the IgG and IgM anti-*T*. *gondii* in human serum, plasma, or whole blood by utilizing *T*. *gondii*-specific antigens. In this study, the serum was used for the detection of IgG and IgM anti-*T*. *gondii* among slaughterhouse workers. Under sterile and aseptic conditions, 3 ml of blood was taken from the slaughterhouse workers by a laboratory assistant. Each specimen tube (red top collection tube) was labeled with the respondent’s identification, kept in the ice-packed cooler, and transported to the laboratory. The test was performed in the Microbiology Laboratory of Defence Services Medical Academy within 24 hours of blood collection. Any materials of human origin were considered infectious and were handled by using standard bio-safety procedures. The blood specimens were allowed to clot and stored at 2–8°C. Serum separation was done after centrifugation of blood samples at 2000 rpm for 10 minutes.

For the accuracy of testing on the *OnSite* Toxo IgG/IgM Combo Rapid Test, the comparison for all subjects showed 98.5% overall agreement for the IgG test line with 91.6% sensitivity and 99.0% specificity and 99.3% overall agreement for the IgM test line with 100% sensitivity and 99.3% specificity. In the serological testing:

**Negative result**: If only the C line developed, the test indicated that anti-*T*. *gondii* antibodies were not detected in the specimen. The result was negative or non-reactive.
**Positive result**: In addition to the presence of the C line, if only the M line was developed, the test indicates the presence of IgM anti-*T*. *gondii*. The result was IgM anti-*T*. *gondii* positive or reactive and IgG anti-*T*. *gondii* negative or non-reactive.
  OR
  In addition to the presence of the C line, if only the G line was developed, the test indicated the presence of IgG anti-*T*. *gondii*. The result was IgG anti-*T*. *gondii* positive or reactive and IgM anti-*T*. *gondii* negative or non-reactive.
  OR
  In addition to the presence of the C line, if both the M and the G lines developed, the test indicated the presence of IgM anti-*T*. *gondii* and IgG anti-*T*. *gondii*. The result was IgM anti-*T*. *gondii* and IgG anti-*T*. *gondii* positive or reactive.

### Data quality control

Before data collection, the principal investigator provided two days of intensive training to four data collectors focusing on the objective of the study, data collection tools and procedures, techniques of interview, and confidentiality of information. A senior public health specialist and a microbiologist were involved as the supervisors who made daily supervision of the collected data throughout the study period. The data were also carefully checked for completeness, clarity, and consistency. Any confusion on the data collection procedure and responses were handled timely.

### Statistical analysis

Collected data were checked for completeness and inconsistencies. Then the data were entered into Microsoft Excel sheet version 2016 and were analyzed by IBM SPSS Statistics for Windows, Version 23.0 (Armonk, NY: IBM Corp). To illustrate the descriptive findings, categorical variables were summarized as numbers and percentages. Mean and standard deviation were expressed for continuous variables. The normality of the knowledge score was viewed by using the Kolmogorov-Smirnov test and the data distribution was non-normal. Therefore, the level of knowledge was categorized as low (< 5) and high (≥ 5) by the median cutoff point. Bivariate logistic regression analysis was performed to find the risk factors of *T*. *gondii* seropositivity in slaughterhouse workers. The strength of associations was presented with an odds ratio (OR) with the corresponding 95% confidence interval (CI). A level of statistical significance was declared at a p value <0.05.

### Ethical consideration

Ethical clearance was obtained from the Ethical Review Committee, Defence Services Medical Academy, Yangon Region, Myanmar and a letter of permission to conduct the study was obtained from the administrative person of YCDC. Before data collection, the purpose of the study was clearly described to the study participants including the benefits and risks of the study. After the sufficient explanation, written informed consent was obtained from all participants. Any information concerning the participants was kept confidential and the specimen collected from the participants was only analyzed for the intended purposes. The participants with seropositivity of *T*. *gondii* were communicated with the respective health professionals of No.1 Defence Services General Hospital (1000-bedded) for possible interventions.

## Results

Overall, 139 slaughterhouse workers in Yangon Region were included in the analysis. Among the participants, 85.6% were males and 14.4% were females. The mean (±SD) age of the participants was 32.97 (±11.96) years with the range of 18–66 years and 54.0% were older than 30 years. Of all participants, 25.2% were a high school education and above, 43.9% of living with five and more family members, 15.1% of residing in rural areas, and 7.2% of blood transfusion history. A total of 41.0% had soil contact, 19.4% ate raw meat, and 46.0% had a low level of knowledge regarding toxoplasmosis. As the occupational factors, there were 56.8% of participants with longer than three years of service duration, 88.5% of contact with animal organs, muscles, or blood, 54.7% of work-related accidents, and 84.9% of exposed to animals depending on their jobs. Among the participants exposed to animals, 40.7%, 38.1%, and 21.2% frequently slaughtered goats, pigs, and cattle respectively.

Regarding the environmental factors, 28.8% of the participants responded to having cats at their houses. Of all participants, there were 79.1% of treated water supply usage, 84.2% covering the water tanks, 66.2% of septic tanks in sewage disposal, 60.4% of open dumping for garbage disposal, and 61.2% of rodents in their houses. In this study, 43.9% (95% CI: 35.5–52.5%) of participants were seropositive for *T*. *gondii* antibodies (IgG and/or IgM), of whom 98.4% (95% CI: 91.2–100.0%) were positive for IgG antibodies and 1.6% (95% CI: 0.0–8.8%) was positive for both IgG and IgM antibodies (Table 1).

**Table 1:** Seropositivity of *Toxoplasma gondii* among slaughterhouse workers.

Seropositivity	n (%)
Positive	61 (43.9)
IgG positive	60 (98.4)
Both IgG and IgM positive	1 (1.6)
Negative	78 (56.1)

In binary logistic regression analysis, the four factors were associated with increased odds of *T*. *gondii* infection. The associated socioeconomic factors of *T*. *gondii* seropositivity are shown in Table 2. The participants reporting a history of blood transfusion were higher in *T*. *gondii* seropositivity than those who had not (OR: 5.74, 95% CI: 1.17–28.09). The participants with a low level of knowledge regarding toxoplasmosis were more likely to get *T*. *gondii* infection than those with a high level of knowledge (OR: 2.91, 95% CI: 1.46–5.83). As shown in Table 3, the participants who contact with animal organs, muscles, and blood also had higher odds of seropositivity for *T*. *gondii* antibodies than their counterparts (OR: 14.29, 95% CI: 1.83–111.51). Among the participants exposed to animals (n = 118), the participants who frequently slaughtered cattle were increased odds of being seropositive than those who slaughtered pigs (OR: 3.22, 95% CI: 1.16–8.93). There was no associated environmental factor with the seropositivity of *T*. *gondii* among slaughterhouse workers (Table 4).

**Table 2:** Sociodemographic characteristics and level of knowledge associated with seropositivity of *Toxoplasma gondii* among slaughterhouse workers.

Sociodemographic characteristics	Total n (%)	Seropositive n (%)	OR (95% CI)	p value
Sex				
Female	20 (14.4)	8 (40.0)	1	
Male	119 (85.6)	53 (44.5)	1.21 (0.46–3.16)	0.705
Age (year)				
≤ 30	64 (46.0)	26 (40.6)	1	
> 30	75 (54.0)	35 (46.7)	1.28 (0.65–2.51)	0.475
Education				
High school education and above	35 (25.2)	11 (31.4)	1	
Middle school education and below	104 (74.8)	50 (48.1)	2.02 (0.89–4.54)	0.089
Marital status				
Single	42 (30.2)	15 (35.7)	1	
Married and others ^⁑^	97 (69.8)	46 (47.4)	1.62 (0.77–3.43)	0.203
Family member				
< 5	78 (56.1)	30 (38.5)	1	
≥ 5	61 (43.9)	31 (50.8)	1.65 (0.84–3.25)	0.146
Monthly family income (kyat)				
> 300,000	42 (30.2)	17 (40.5)	1	
≤ 300,000	97 (69.8)	44 (45.4)	1.22 (0.59–2.55)	0.594
Residence				
Rural	21 (15.1)	7 (33.3)	1	
Urban	118 (84.9)	54 (45.8)	1.69 (0.64–4.48)	0.294
History of blood transfusion				
No	129 (92.8)	53 (41.1)	1	
Yes	10 (7.2)	8 (80.0)	5.74 (1.17–28.09)	0.031
Soil contact				
No	82 (59.0)	36 (43.9)	1	
Yes	57 (41.0)	25 (43.9)	1.00 (0.51–1.97)	0.998
Eating raw meat				
No	112 (80.6)	48 (42.9)	1	
Yes	27 (19.4)	13 (48.1)	1.24 (0.53–2.88)	0.619
Level of knowledge				
High	75 (54.0)	24 (32.0)	1	
Low	64 (46.0)	37 (57.8)	2.91 (1.46–5.83)	0.003

^⁑^ married and others–the participants who were married, widowed, divorced, and separate

**Table 3:** Occupational factors associated with seropositivity of *Toxoplasma gondii* among slaughterhouse workers.

Occupational factors	Total n (%)	Seropositive n (%)	OR (95% CI)	p value
Duration of work at current sector (year)				
≤ 3	60 (43.2)	25 (41.7)	1	
> 3	79 (56.8)	36 (45.6)	1.17 (0.59–2.31)	0.646
Receive training before starting to work				
No	124 (89.2)	54 (43.5)	1	
Yes	15 (10.8)	7 (46.7)	1.13 (0.39–3.32)	0.818
Contact with animal organs, muscles or blood				
No	16 (11.5)	1 (6.3)	1	
Yes	123 (88.5)	60 (48.8)	14.29 (1.83–111.51)	0.011
Work-related accidents				
No	63 (45.3)	31 (49.2)	1	
Yes	76 (54.7)	30 (39.5)	0.67 (0.34–1.32)	0.251
Job in slaughterhouse				
Office work	21 (15.1)	6 (28.6)	1	
Exposure to animals ^⁑^	118 (84.9)	55 (46.6)	2.18 (0.79–6.01)	0.131
Animals most frequently slaughtered (n = 118)				
Pig	45 (38.1)	16 (35.6)	1	
Goat	48 (40.7)	23 (47.9)	1.67 (0.73–3.83)	0.229
Cattle	25 (21.2)	16 (64.0)	3.22 (1.16–8.93)	0.024
Personal protective wearing (n = 118)				
Yes	48 (40.7)	18 (37.5)	1	
No	70 (59.3)	37 (52.9)	1.87 (0.88–3.95)	0.102
Smoke during work break (n = 118)				
No	69 (58.5)	31 (44.9)	1	
Yes	49 (41.5)	24 (49.0)	1.18 (0.57–2.45)	0.664
Wash hand before and after eating (n = 118)				
Yes	101 (85.6)	47 (46.5)	1	
No	17 (14.4)	8 (47.1)	1.02 (0.37–2.86)	0.968

^⁑^ Exposure to animals–the participants who were working in slaughter rooms, inspection rooms, cauldron, and meat delivery

**Table 4:** Environmental factors associated with seropositivity of *Toxoplasma gondii* among slaughterhouse workers.

Environmental factors	Total n (%)	Seropositive n (%)	OR (95% CI)	p value
Presence of cats at house				
No	99 (71.2)	43 (43.4)	1	
Yes	40 (28.8)	18 (45.0)	1.067 (0.51–2.23)	0.866
Treated water supply				
Yes	110 (79.1)	48 (43.6)	1	
No	29 (20.9)	13 (44.8)	1.05 (0.46–2.39)	0.570
Water tank cover				
Yes	117 (84.2)	52 (44.4)	1	
No	22 (15.8)	9 (40.9)	0.87 (0.34–2.28)	0.759
Sewage				
Septic tank	92 (66.2)	40 (43.5)	1	
Public collection system	47 (33.8)	21 (44.7)	1.05 (0.52–2.13)	0.892
Garbage disposal				
Rubbish bins	51 (36.7)	22 (43.1)	1	
Open dumping	84 (60.4)	38 (45.2)	1.09 (0.54–2.19)	0.812
Refuse pits	4 (2.9)	1 (25.0)	0.44 (0.04–4.52)	0.489
Vacant lots next to the house				
No	87 (62.6)	38 (43.7)	1	
Yes	52 (37.4)	23 (44.2)	1.02 (0.51–2.04)	0.949
Flooded areas next to the house				
No	81 (58.3)	31 (38.3)	1	
Yes	58 (41.7)	30 (51.7)	1.73 (0.87–3.42)	0.116
Rodents in house				
No	54 (38.8)	19 (35.2)	1	
Yes	85 (61.2)	42 (49.4)	1.79 (0.89–3.63)	0.101

## Discussion

Toxoplasmosis, a significant public health problem worldwide, may be either acute or chronic and can cause active infection at any age [55]. Myanmar, a developing country, is still having limitations in the detection of *T*. *gondii* in persons who contact with animals, as occupationally. The serological test was performed to detect the IgG for convalescent infection, IgM for recent or active infection, and their combination. The overall prevalence of antibodies for *T*. *gondii* observed in this study was 43.9% and it was higher than the findings of the studies done in Thailand 1.5% [26], China 11.6% [39], and Japan 32.8% [32]. However, it was lower than those reported in the previous studies that were conducted among butchers [56], abattoirs [19, 31, 57], and slaughterhouse workers [36, 58], ranging from 50.0% to 72.0%. The IgG was positive in 43.2% (60 out of 139) of slaughterhouse workers in this study. It was higher than IgG positive of the study done in Makkah 25.0% [40] and lower than the findings of the studies conducted in Nigeria 55.8% [34], Brazil 59.5% [30], Saudi Arabia 80.0% [33], and Kenya 83.9% [42]. These seroprevalence variations could be due to differences in the geographical background of the study population, weather conditions, socioeconomic factors (such as educational level and job nature), personal hygiene, feeding habits, and contact with cats. Additionally, differences in serological methods used (latex-agglutination test, microscopical seroagglutination test, indirect fluorescence antibody test, and enzyme-linked immunoassay) and sensitivity of these methods were other explanations for such discrepancy [32, 36, 39, 40]. Most studies were diagnosed using enzyme-linked immunoassay due to the rapidity and convenience, the accuracy of the method, and cost-effectiveness.

In this study, history of blood transfusion, level of knowledge about toxoplasmosis, contact with animal organs, muscles, or blood, and animals most frequently slaughtered (cattle) were identified as the risk factors significantly associated with *T*. *gondii* infection among slaughterhouse workers. The presence of a history of blood transfusion was an associated factor of *T*. *gondii* infection among slaughterhouse workers. It supported the finding of a seroprevalence study that a history of blood transfusion was a risk factor for *T*. *gondii* infection [59]. Therefore, it provided the suggestion that blood for anti-*T*. *gondii* antibodies should be undertaken as a pre-transfusion screening because transmission of toxoplasmosis from the blood is a possibility when the donor was infected.

The knowledge of transmission pathways and precautions to take had optimistic effects on the prevention and control of infectious disease transmission. In this study, the level of knowledge about toxoplasmosis and seropositivity were associated among slaughterhouse workers. There was nearly half of the low knowledge about toxoplasmosis, including the route of transmission, clinical manifestations, and preventive measures of *T*. *gondii* infection. Moreover, there were only 10.8% of slaughterhouse workers received training in occupational safety before starting their work and it might be the insufficient knowledge of preventive measures in their workplaces.

The slaughterhouse workers can be considered a high-risk group for toxoplasmosis for their contact with raw meat, animals’ blood, and organs [42]. In this study, contact with animal organs, muscles, or blood was a risk factor for *T*. *gondii* infection among slaughterhouse workers. This finding was consistent with the result of a previous study done among high-risk populations in China stated that the livestock breeding/processing staff were at higher risk for *T*. *gondii* infection due to a frequently contact with animals and animal products [39]. However, it was contradicted by the finding of the study conducted in Brazil demonstrated that direct contact with blood or organs in the slaughterhouse was not associated with *T*. *gondii* infection [36].

Furthermore, there was an association between animals most frequently slaughtered and the seropositivity of *T*. *gondii* in this study. It was in agreement with the previous studies done in Nigeria and China showed that types of slaughtered animal exposure were significantly associated with seropositivity of toxoplasmosis [34, 39]. However, this result had been unable to demonstrate in Kenya study, showing that the slaughtered animal types were not associated with seropositivity to *T*. *gondii* [42].

In this study, sex, age, educational level, and residence were not associated with toxoplasmosis among slaughterhouse workers. The previous studies done in Brazil and Kenya also expressed that there was no association between sex and seroprevalence of toxoplasmosis [36, 42]. However, in contrast to this study, evidence of a significant association between age and *T*. *gondii* infection was detected in the previous studies [32, 34, 40, 42, 57]. Exposure to sporulated oocysts-contaminated soil and consumption of bradyzoites from raw or undercooked meat were the major horizontal routes of transmission [60]. However, the findings of the current study do not support them and other studies also found that the seropositivity of *T*. *gondii* was not associated with eating raw milk or meat and contact with soil [30, 34, 36]. Moreover, in this study, the presence of cats and rodents at house was not significantly associated with toxoplasmosis among slaughterhouse workers. These findings matched those observed in earlier studies [30, 34, 36].

There were some limitations in this study. First, a limited study population of workers involving in the main slaughterhouses of Yangon Region and a small sample size made generalization based on the observed result very difficult. A significant imbalance in sex was also occurred in this study. The results of this study should be interpreted carefully because the fewer recruits would reduce power and make the non-significant results more likely, even if in a reality there were true associations. Second, it was not possible to infer causality between *T*. *gondii* infection and the significant predictors due to a cross-sectional design. Third, only seroprevalence was applied to detect the positive slaughterhouse workers and the genotypic characterization of *T*. *gondii* strains could not be detected in this study.

## Conclusions

This is the first study to assess the seroprevalence of *T*. *gondii* and its associated factors among the slaughterhouse workers in Yangon Region, Myanmar and 43.9% of the study population were seropositive for *T*. *gondii* infection. History of blood transfusion, level of knowledge about toxoplasmosis, contact with animal organs, muscles, or blood, and animals most frequently slaughtered (cattle) were identified as possible risk factors associated with *T*. *gondii* infection. There is a need for preventive measures such as providing health education regarding transmission and preventive measures for their safety at workplaces, enforcement of training for personal hygiene practices in workplaces, the commencement of the serological screening or formal risk assessment on both slaughtered animals and slaughterhouse workers, and developing the surveillance system to control the toxoplasmosis.

## Supporting information

S1 File English version of the questionnaire. (PDF)

S2 File Myanmar version of the questionnaire. (PDF)

S3 File Minimal data. (XLSX)
