# Supplementary material for: Seroprevalence and associated risk factors of Toxoplasma gondii infection among slaughterhouse workers in Yangon Region, Myanmar: A cross-sectional study
Source: PLoS One. 2023 Apr 13;18(4):e0284352. doi: 10.1371/journal.pone.0284352 (PMC10101469; doi:10.1371/journal.pone.0284352)
Supplement: S1 File — (PDF) [file pone.0284352.s001.pdf]

Questionnaire (English)

Code No. ___/___/___

**Sociodemographic characteristics**			
Age ________________________ years		Sex	☐ Male ☐ Female
Education	☐ Illiterate<br>☐ Primary school level<br>☐ Middle school level<br>☐ High school level<br>☐ University or College<br>☐ Graduate	Marital status	☐ Single<br>☐ Married<br>☐ Widowed<br>☐ Divorced<br>☐ Separate
Family members ___________________		Monthly family income _________________kyat	
Residence	☐ Rural<br>☐ Urban	Blood transfusion history	☐ Yes<br>☐ No
Soil contact	☐ Yes<br>☐ No	Eating raw meat	☐ Yes<br>☐ No

**Knowledge about toxoplasmosis**
1. Can working in contact with raw meat cause parasitic infections?<br>☐ Yes ☐ No ☐ Don’t know
2. Can cat transmit *Toxoplasma* infection?<br>☐ Yes ☐ No ☐ Don’t know
3. Can *Toxoplasma* infection be transmitted by consumption of contaminated food or drinks?<br>☐ Yes ☐ No ☐ Don’t know
4. Can Toxoplasmosis infection be transmitted by consumption of raw meat?<br>☐ Yes ☐ No ☐ Don’t know
5. Can *Toxoplasma* be in meat we eat?<br>☐ Yes ☐ No ☐ Don’t know
6. Can *Toxoplasma* be inactivated by freezing meat?<br>☐ Yes ☐ No ☐ Don’t know
7. Can *Toxoplasma* infection be transmitted by consumption of unboiled or untreated water?<br>☐ Yes ☐ No ☐ Don’t know
8. Can *Toxoplasma* infection be transmitted by consumption of unwashed fruits or vegetables?<br>☐ Yes ☐ No ☐ Don’t know
9. Can *Toxoplasma* infection be transmitted by organ or tissue transplantation?<br>☐ Yes ☐ No ☐ Don’t know
10. Can *Toxoplasma* infection be transmitted by blood transfusion?<br>☐ Yes ☐ No ☐ Don’t know
11. Can *Toxoplasma* be present in cat’s faeces?<br>☐ Yes ☐ No ☐ Don’t know
12. Can *Toxoplasma* be present in soil?<br>☐ Yes ☐ No ☐ Don’t know
13. Can *Toxoplasma* cause miscarriage?<br>☐ Yes ☐ No ☐ Don’t know
14. Can *Toxoplasma* cause disease in fetus?<br>☐ Yes ☐ No ☐ Don’t know
15. Can *Toxoplasma* cause eye diseases?<br>☐ Yes ☐ No ☐ Don’t know
16. Is there any treatment for toxoplasmosis?<br>☐ Yes ☐ No ☐ Don’t know
17. Do you know someone with toxoplasmosis in your surroundings?<br>☐ Yes ☐ No ☐ Don’t know

**Occupational factors**
1. Duration of work in current sector _________________________ years
2. Did you receive any training before you start working?<br>☐ Yes ☐ No
3. Do you have any contact with animal organs, muscles, blood?<br>☐ Yes ☐ No
4. Have you had work related accident?<br>☐ Yes ☐ No
5. What is your job in slaughterhouse?<br>☐ Office work ☐ Exposure to animals
If expose to animals, ask the following:
6. What animals are most frequently slaughtered?<br>☐ Pig ☐ Goat ☐ Cattle<br>Others (please specify) ____
7. Do you wear the personal protective wearing (PPE)?<br>☐ Yes ☐ No
8. Do you smoke during your work breaks?<br>☐ Yes ☐ No
9. Do you wash your hands before and after eating?<br>☐ Yes ☐ No

**Environmental factors**		
1. Do you have cats at house?		
☐ Yes	☐ No	
2. Does the house have treated water supply?		
☐ Yes	☐ No	
3. Is the water tank covered?		
☐ Yes	☐ No	
4. Sewage system		
☐ Public collection system	☐ Septic tank	☐ Pit latrine
Others (please specify) ______________________________		
5. Garbage disposal		
☐ Rubbish bins	☐ Refuse pits	☐ Open dumping
Others (please specify) ______________________________		
6. Are the vacant lots next to the house?		
☐ Yes	☐ No	
7. Is there flooded area next to the house?		
☐ Yes	☐ No	
8. Are the rodents found in the house?		
☐ Yes	☐ No	
