# Supplementary material for: Seroprevalence and associated risk factors of Toxoplasma gondii infection among slaughterhouse workers in Yangon Region, Myanmar: A cross-sectional study
Source: PLoS One. 2023 Apr 13;18(4):e0284352. doi: 10.1371/journal.pone.0284352 (PMC10101469; doi:10.1371/journal.pone.0284352)
Supplement: S2 File — (PDF) [file pone.0284352.s002.pdf]

Questionnaire (Myanmar)

Code No. ___/___/___

ပုဂ္ဂိုလ်ရေးဆိုင်ရာ အချက်အလက်များ			
အသက်(ပြည့်ပြီးအသက်) ______________ နှစ်		ကျား/မ	☐ ကျား ☐ မ
ပညာအရည်အချင်း	☐ စာမတတ်<br>☐ မူလတန်းပညာ<br>☐ အလယ်တန်းပညာ<br>☐ အထက်တန်းပညာ<br>☐ တက္ကသိုလ်/ကောလိပ်ပညာ<br>☐ ဘွဲ့ရ	အိမ်ထောင်ရေးအခြေအနေ	☐ လူပျို/အပျို<br>☐ အိမ်ထောင်ရှိ<br>☐ မုဆိုးဖို/မ<br>☐ အိမ်ထောင်ကွဲ<br>☐ မတူမနေ
အိမ်ထောင်စုဝင်အရေအတွက် __________________		အိမ်ထောင်စုလစဉ်ဝင်ငွေ ______________ ကျပ်	
နေထိုင်ရာအရပ်	☐ ကျေးလက်<br>☐ မြို့ပြ	သွေးသွင်းခံရဖူးခြင်း	☐ သွင်းဖူးပါသည်<br>☐ မသွင်းဖူးပါ
မြေကြီးနှင့် ထိစပ်သောအလုပ် လုပ်ခဲ့ဖူးခြင်း	☐ လုပ်ဖူးပါသည်<br>☐ မလုပ်ဖူးပါ	အသားစိမ်း/မကျက်တကျက် များကိုစားသုံးခြင်း	☐ စားသုံးပါသည်<br>☐ မစားသုံးပါ

Toxoplasmosis ဗဟုသုတဆိုင်ရာမေးခွန်းများ		
၁။ အသားစိမ်းများနှင့်ထိတွေ့၍ အလုပ်လုပ်ရခြင်းကြောင့် **Toxoplasmosis** ကပ်ပါးရောဂါတစ်မျိုး ဖြစ်စေနိုင်ပါသလား။		
☐ ဖြစ်နိုင်ပါသည်	☐ မဖြစ်နိုင်ပါ	☐ မသိပါ
၂။ ကြောင်သည် အဆိုပါကပ်ပါးရောဂါကို ပြန့်ပွားစေနိုင်ပါသလား။		
☐ ပြန့်ပွားစေနိုင်ပါသည်	☐ မပြန့်ပွားနိုင်ပါ	☐ မသိပါ
၃။ အဆိုပါကပ်ပါးရောဂါသည် ရောဂါထိတွေ့ထားသောအစာနှင့်ရေကို သောက်စားခြင်းကြောင့် ကူးစက်နိုင်ပါသလား။		
☐ ကူးစက်နိုင်သည်	☐ မကူးစက်နိုင်ပါ	☐ မသိပါ
၄။ အဆိုပါ ကပ်ပါးရောဂါသည် အသားစိမ်းများစားခြင်းကြောင့် ကူးစက်နိုင်ပါသလား။		
☐ ကူးစက်စေနိုင်သည်	☐ မကူးစက်နိုင်ပါ	☐ မသိပါ
၅။ ကျွန်ုပ်တို့စားသောအသားများတွင် အဆိုပါ ကပ်ပါးရောဂါပိုး ပါရှိနေပါသလား။		
☐ ထင်ပါသည်	☐ မထင်ပါ	☐ မသိပါ
၆။ အဆိုပါ ကပ်ပါးရောဂါပိုးကို အသားများအား အေးခဲစေခြင်းဖြင့် နှိမ်နင်းနိုင်ပါသလား။		
☐ နှိမ်နင်းနိုင်သည်	☐ မနှိမ်နင်းနိုင်ပါ	☐ မသိပါ
၇။ အဆိုပါ ကပ်ပါးရောဂါပိုးသည် သန့်စင်ထားခြင်းမရှိသော သို့မဟုတ် မကျိုချက်ထားသော သောက်ရေမှ တဆင့် ကူးစက်နိုင်ပါသလား။		
☐ ကူးစက်နိုင်သည်	☐ မကူးစက်နိုင်ပါ	☐ မသိပါ
၈။ အဆိုပါ ကပ်ပါးရောဂါပိုးသည် မဆေးကြော ထားသော အသီးရွက် သို့မဟုတ် အသီးအနှံများ စားသုံးခြင်း ဖြင့် ကူးစက်နိုင်ပါသလား။		
☐ ကူးစက်နိုင်သည်	☐ မကူးစက်နိုင်ပါ	☐ မသိပါ
၉။ အဆိုပါ ကပ်ပါးရောဂါပိုးသည် ခန္ဓာကိုယ် အစိတ်ပိုင်းများ လဲလှယ်ကုသခြင်းမှတဆင့် ကူးစက်နိုင်ပါ သလား။		
☐ ကူးစက်နိုင်သည်	☐ မကူးစက်နိုင်ပါ	☐ မသိပါ
၁၀။ အဆိုပါ ကပ်ပါးရောဂါပိုးသည် သွေးသွင်းခြင်း မှတဆင့် ကူးစက်နိုင်ပါသလား။		
☐ ကူးစက်နိုင်သည်	☐ မကူးစက်နိုင်ပါ	☐ မသိပါ
၁၁။ အဆိုပါ ကပ်ပါးရောဂါပိုးကို ကြောင်၏မစင်တွင် တွေ့နိုင်ပါသလား။		
☐ တွေ့နိုင်ပါသည်	☐ မတွေ့နိုင်ပါ	☐ မသိပါ
၁၂။ အဆိုပါ ကပ်ပါးရောဂါပိုးကို မြေကြီးတွင် တွေ့နိုင်ပါသလား။		
☐ တွေ့နိုင်ပါသည်	☐ မတွေ့နိုင်ပါ	☐ မသိပါ
၁၃။ အဆိုပါ ကပ်ပါးရောဂါပိုးကြောင့် ကိုယ်ဝန် ပျက်ကျ နိုင်ပါသလား။		
☐ ပျက်ကျနိုင်ပါသည်	☐ မပျက်ကျနိုင်ပါ	☐ မသိပါ

၁၄။ အဆိုပါ ကပ်ပါးရောဂါပိုးကြောင့် သန္ဓေသား သေဆုံးနိုင်ပါသလား။<br>☐ သေဆုံးနိုင်သည် ☐ မသေဆုံးနိုင်ပါ ☐ မသိပါ
၁၅။ အဆိုပါ ကပ်ပါးရောဂါပိုးကြောင့် မျက်စိရောဂါများ ဖြစ်ပွားနိုင်ပါသလား။<br>☐ ဖြစ်ပွားနိုင်သည် ☐ မဖြစ်ပွားနိုင်ပါ ☐ မသိပါ
၁၆။ အဆိုပါ ကပ်ပါးရောဂါကို ကုသနိုင်သော နည်းလမ်းများရှိပါသလား။<br>☐ ရှိပါသည် ☐ မရှိပါ ☐ မသိပါ
၁၇။ အဆိုပါ ကပ်ပါးရောဂါဖြစ်ဖူးသော ပုဂ္ဂိုလ်တစ်ဦးဦးအား သင်၏ပတ်ဝန်းကျင်တွင် တွေ့ဖူးပါသလား။<br>☐ တွေ့ဖူးပါသည် ☐ မတွေ့ဖူးပါ ☐ မသိပါ

လုပ်ငန်းခွင်ဆိုင်ရာ အချက်အလက်များ
၁။ လက်ရှိနေရာတွင် လုပ်သက် (ပြည့်ပြီးနှစ်) ______________________ နှစ်
၂။ လုပ်ငန်းခွင်မဝင်ခင်တွင် အလုပ်နှင့် ပတ်သက်သောသင်တန်းများ ရရှိခဲ့ဖူးပါသလား။<br>☐ ရရှိဖူးပါသည် ☐ မရရှိဖူးပါ
၃။ သင်သည် တိရစ္ဆာန် ခန္ဓာကိုယ်အစိတ်ပိုင်း၊ အသားနှင့် သွေးများဖြင့် ထိတွေ့မှုရှိပါသလား။<br>☐ ရှိပါသည် ☐ မရှိပါ
၄။ အလုပ်နှင့် ဆက်စပ်၍ ထိခိုက်ဒဏ်ရာ ရရှိဖူးပါသလား။<br>☐ ရရှိဖူးပါသည် ☐ မရရှိဖူးပါ
၅။ သားသတ်ရုံတွင် လုပ်ကိုင်ရသောနေရာ<br>☐ ရုံးလုပ်ငန်း/စာရင်းကိုင် ☐ တိရစ္ဆာန်များနှင့်ထိတွေ့ရသော အလုပ်
တိရစ္ဆာန်များနှင့်ထိတွေ့ရသော အလုပ်ဖြစ်ပါက:
၆။ အများအားဖြင့် သားသတ်ခြင်းလုပ်ရသောတိရစ္ဆာန်များ<br>☐ ဝက် ☐ ဆိတ် ☐ နွား/အမဲ<br>အခြားရှိပါက ဖော်ပြရန် ______________________________
၇။ သင်သည် လုပ်ငန်းခွင်တွင် အကာအကွယ်များ ဝတ်ဆင်ပါသလား။<br>☐ ဝတ်ဆင်ပါသည် ☐ မဝတ်ဆင်ပါ
၈။ အလုပ်နားချိန်များတွင် ဆေးလိပ် သောက်တတ်ပါသလား။<br>☐ သောက်ပါသည် ☐ မသောက်ပါ
၉။ အစားအသောက် မသုံးဆောင်ခင်နှင့် သုံးဆောင်ပြီး ချိန်များတွင် လက်ဆေးလေ့ရှိပါသလား။<br>☐ ဆေးပါသည် ☐ မဆေးပါ

ပတ်ဝန်းကျင်ဆိုင်ရာ အချက်အလက်များ
၁။ အိမ်တွင် ကြောင်များမွေးထားပါသလား။<br>☐ မွေးထားပါသည် ☐ မမွေးထားပါ
၂။ အိမ်တွင် သန့်စင်ထားသောရေ သုံးစွဲပါသလား။<br>☐ သုံးစွဲပါသည် ☐ မသုံးစွဲပါ
၃။ ရေစည်အား ဖုံးပိတ်ထားပါသလား။<br>☐ ဖုံးထားပါသည် ☐ မဖုံးပါ
၄။ မည်သည့် မိလ္လာစနစ် သုံးစွဲပါသလဲ။<br>☐ အစိုးရမိလ္လာစနစ် ☐ မိလ္လာကန် ☐ တွင်းအိမ်သာ<br>အခြားရှိပါက ဖော်ပြရန် ______________________________
၅။ မည်သည့် အမှိုက်သိမ်းစနစ် အသုံးပြုပါသလဲ။<br>☐ အမှိုက်ပုံးတွင်ပစ် ☐ သတ်မှတ်အမှိုက်ပုံတွင်ပစ် ☐ ကွင်းပြင်တွင်ပစ်<br>အခြားရှိပါက ဖော်ပြရန် ______________________________
၆။ အိမ်ဘေးတွင် မြေကွက်လပ်ရှိပါသလား။<br>☐ ရှိပါသည် ☐ မရှိပါ
၇။ အိမ်နီးတစ်ဝိုက်တွင် ရေလွှမ်းသောနေရာ ရှိပါသလား။<br>☐ ရှိပါသည် ☐ မရှိပါ
၈။ အိမ်တွင် ကြွက်များ ရှိပါသလား။<br>☐ ရှိပါသည် ☐ မရှိပါ
